# Reconstruction of a traumatic duodenal transection with a pedicled ileal loop: a case report

**DOI:** 10.1186/1752-1947-4-343

**Published:** 2010-10-26

**Authors:** Apostolos Kambaroudis, Nikolaos Antoniadis, Savvas Papadopoulos, Charalambos Spiridis, Thomas Gerasimidis

**Affiliations:** 1 5th Surgical Clininc, Hippokrateion General Hospital, 49 Konstantinoupoleos str., P.O. 54642, Thessaloniki, Greece

## Abstract

**Introduction:**

Blunt duodenal injuries do not occur often. A patient with damage to the duodenal tissue around the pancreatic and common bile duct presents a challenge to surgeons. The choice of procedure must be tailored to the nature of the defect and the amount of tissue lost.

**Case presentation:**

We describe the case of a 16-year-old Caucasian boy with a blunt duodenal injury after a motor vehicle accident. On admission, the patient had stable vital signs and a normal laboratory workup. Gradually his clinical condition deteriorated and a computed tomography scan showed a retroperitoneal haematoma at the level of his duodenum. A fully circumferential rupture of the second part of his duodenum was found during laparotomy, with the intact Vater's papilla lying adjacent to the defect and a superficial laceration of the head of his pancreas. The retroperitoneal haematoma was thoroughly drained and a pedicled ileal loop was interposed between the duodenal stumps to restore the continuity of the patient's duodenum. Apart from a mild postoperative pancreatitis, the patient's postoperative course evolved with no further problems. The patient was discharged on the 22^nd ^postoperative day in excellent condition and has remained so to date (after five years).

**Conclusion:**

In our case report, where the second part of the patient's duodenum was completely transected, our choices for reconstruction were limited. Important factors for the successful management of this patient were prompt surgical intervention and the accurate assessment of the nature of the duodenal and associated injuries. We believe that the technique we used was a reasonable choice because the anatomical continuity of the patient's duodenum was restored.

## Introduction

Patients with duodenal injuries represent approximately 4% of all patients with abdominal injuries from blunt trauma, usually resulting from motor vehicle accidents, which account for 22% of all patients with duodenal injuries [1]. Due to the anatomical position of the duodenum, blunt duodenal trauma is usually associated with injuries to adjacent structures, including the pancreas, bile duct, mesenteric vessels, and inferior vena cava [1]. As the diagnosis of a patient with a blunt duodenal injury is difficult, and even though there are many laboratory tests and radiological studies available, laparotomy with exploration of the retroperitoneal space remains the decisive diagnostic procedure [2]. Delays in diagnosis and treatment result in increased morbidity and mortality, so early diagnosis is very important [3,4].

An array of surgical techniques have been developed for the management of patients with duodenal injuries. The surgeon should choose the most efficient technique according to the type and seriousness of the patient's injury [1].

We describe our case report of a patient with a complete transection of the second part of his duodenum, resulting from a blunt abdominal injury. The surgical technique that was implemented is somewhat different from those that are usually described.

## Case presentation

A 16-year-old Caucasian boy was brought to the emergency department of our hospital after a motor vehicle accident. According to the description of the accident, the young man was hurled from his motorcycle and hit an immobile obstacle, impacting on it with his anterior abdominal wall. He had no apparent external injuries. When he arrived at the hospital he was haemodynamically stable with a blood pressure reading of 120/80 mmHg, a heart rate of 88 pulses/minute and a Glasgow Coma Scale(GCS) score of 15. The patient experienced pain and tenderness on palpation of his right upper abdominal quadrant; the rest of his abdomen was soft and nontender to palpation.

**Figure 1:**
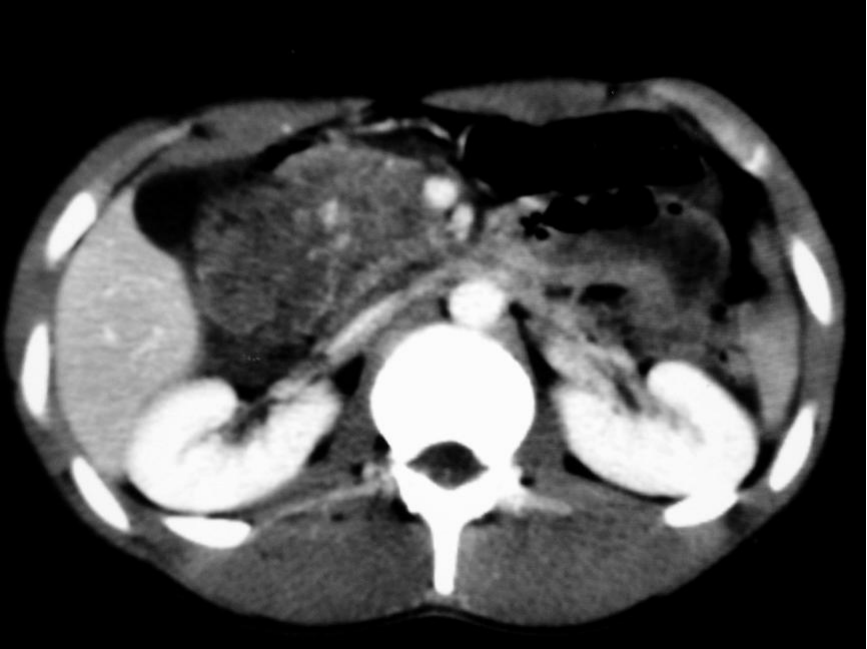
**A computed tomography scan of the patient's abdomen showing a retroperitoneal haematoma at the duodenal level**.

The patient underwent laboratory and radiological examination consisting of x-rays of his head, cervical spine, lumbar spine, chest and abdomen. His blood was cross-matched and an ultrasound examination of his abdominal region was performed in the emergency department to rule out any intra-abdominal haemorrhage and/or any organ injury. Laboratory results showed no specific pathological values (haematocrit of 41% and a white blood cell count of 9,500K/μl). The initial workup did not include serum amylase levels, since a basic serum biochemistry was examined at that time. Plain radiological and ultrasound examinations of the patient showed no pathological findings either. Soon after being admitted to hospital, the patient presented haematemesis and his clinical condition deteriorated. His abdominal pain increased at this time. An abdominal computed tomography (CT) scan without contrast agent administration was subsequently performed. This revealed a retroperitoneal haematoma at the level of the duodenum (Figure 1).

Due to the patient's clinical condition worsening and the CT findings, we did not deem it necessary to perform an upper gastrointestinal endoscopy, and decided to proceed to an immediate exploratory laparotomy. The patient's peritoneal cavity was approached through a midline supra-umbilical incision. No solid organ bleeding or injury was found intraperitoneally. In the region of the head of the pancreas and the second part of the patient's duodenum, there was a retroperitoneal haematoma, which upon investigation was found to contain a fully circumferential rupture of the second part of the duodenum. There was also an apparently superficial rupture of the head of the patient's pancreas.

Both stumps of the patient's injured duodenum were dissected and Vater's papilla was found to be next to the distal stump. The major pancreatic duct was catheterised through the papilla of Vater and saline was injected to check for the presence of a rupture and none was found. The bile duct was also catheterised - as in the case of the pancreatic duct - but no rupture was found along it. Debridement of the stump edges followed, as far as was possible. Due to the position and the extent of the lesion, the risk of disrupting the blood supply of the remaining parts of the patient's duodenum was high and the option of restoration of the duodenal continuity with a primary end-to-end anastomosis was ruled out.

**Figure 2:**
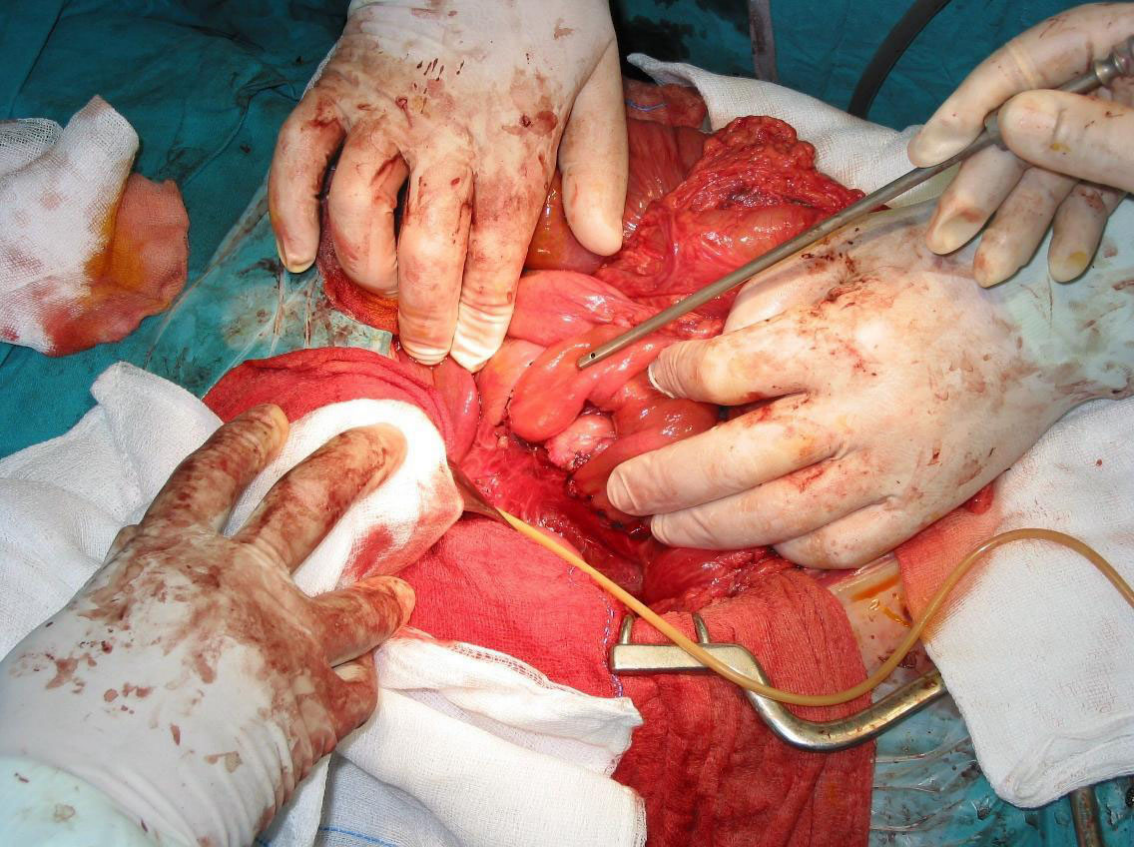
**Two end-to-end anastomoses between the patient's duodenum and pedicled loop of ileum**.

**Figure 3:**
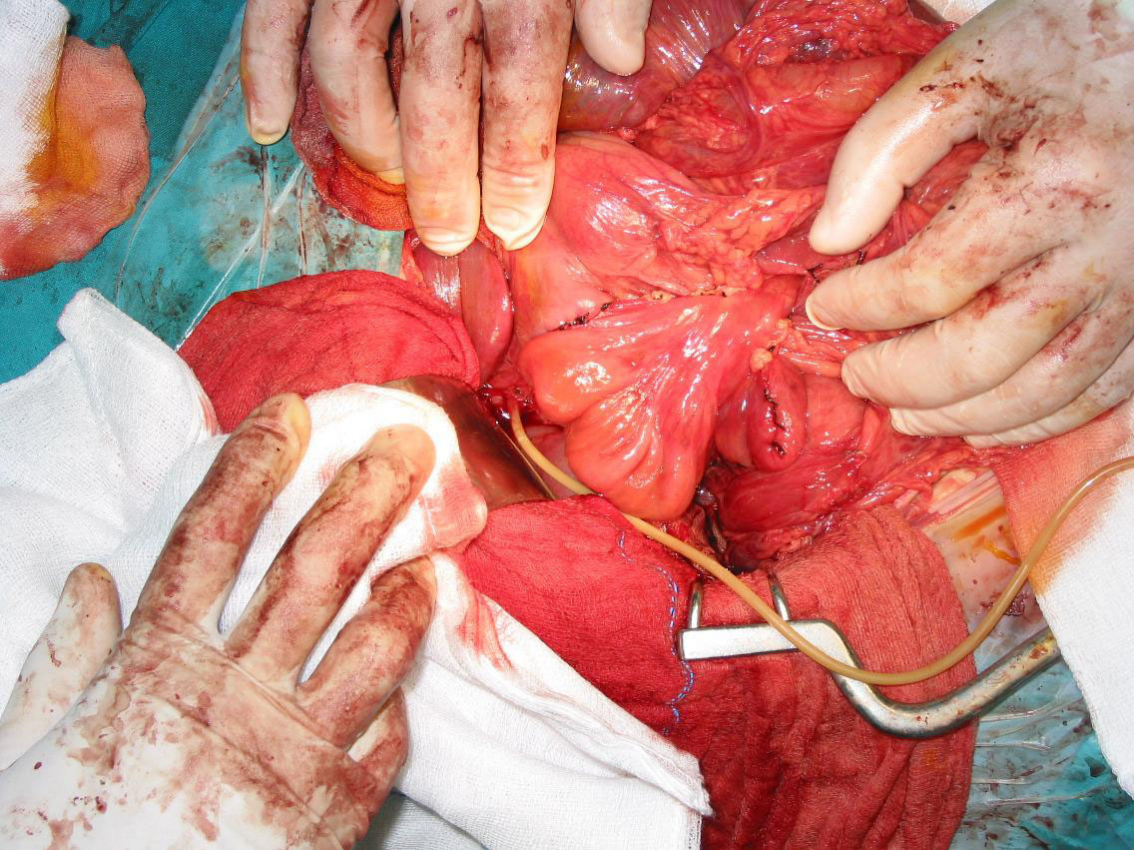
**The pedicled loop of ileum to bridge the duodenal defect**.

In order to restore the continuity of the patient's duodenum, we decided to interpose a pedicled loop of ileum (middle part of ileum) to bridge the gap. The two end-to-end anastomoses were performed at two layers (the bottom one in continuous suture) following catheterisation of Vater's papilla through a choledochotomy so that the papilla could be located and immobilised, in order to avoid including it in the suture line (Figures 2 and 3). Finally, a T-tube was placed through the choledochotomy and an intraoperative cholangiography confirmed that the patient's bile duct was unobstructed and the contrast agent was passing freely into the duodenum. There was no loss of blood during the operation. For better recovery, the patient was transferred to the intensive care unit, where he stayed for five days without presenting any particular problems.

**Figure 4:**
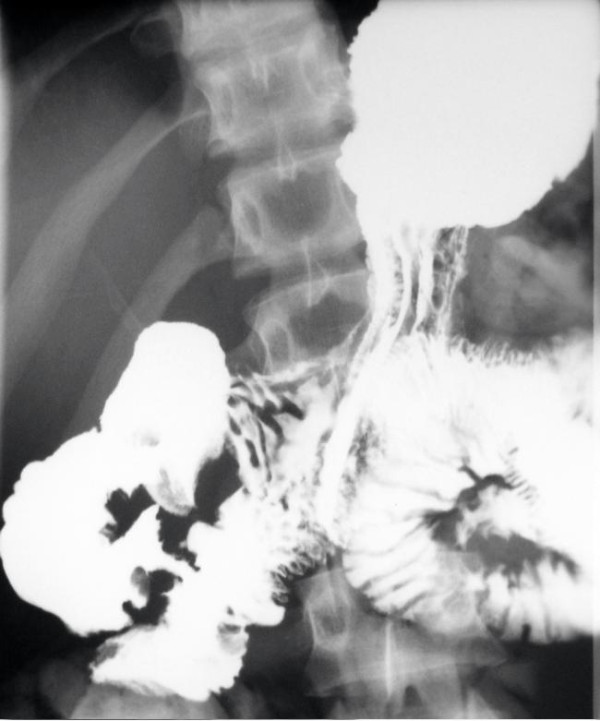
**An upper gastrointestinal contrast study on the 20^th ^postoperative day, without pathological findings**.

**Figure 5:**
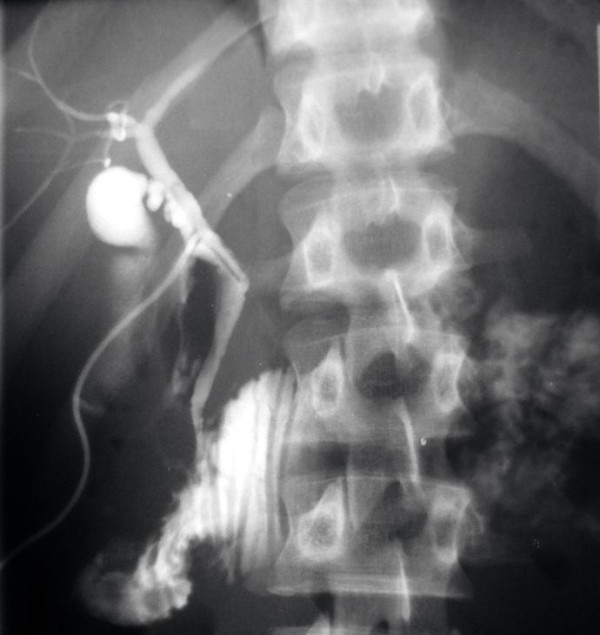
**An intraoperative cholangiography after the reconstruction showing the contrast agent passing freely into the patient's duodenum**.

Postoperatively, the patient was given octreotide subcutaneously at a dosage of 0.1 mg three times a day for a total of 15 days to treat his pancreatic injury. His haematocrit remained stable at about 38%, and his white blood cell count stayed at a steady level of around 10,000 K/μl. His serum amylase level was on average 100 IU/L. On the 10^th ^postoperative day, the patient had mild leukocytosis (17,000 × 10^3^/μl), a serum amylase level of 166 IU/L and a body temperature of up to 38.8°C. An abdominal CT scan showed fluid collection in the region of the head of the patient's pancreas, which was clearly demarcated and not compatible with a pseudocyst. The consensus was that these were manifestations of pancreatitis. The antibiotic treatment was changed from intravenous ampicillin/sulbactam 3 grams once a day to intravenous ciprofloxacin 400 mg two times a day and in the next few days the patient's body temperature dropped and there was a gradual decrease in his white blood cell count and serum amylase levels. The patient continued to be given fluids parenterally. Abdominal CT scans performed on postoperative days 12 and 19 showed a reduction of the fluid in the region of the head of the patient's pancreas and significant improvement of the original imaging findings. The patient was orally fed from the 14^th ^postoperative day and tolerated this very well. An upper gastrointestinal series with water-soluble contrast medium (Gastrografin) was performed on the 20^th ^postoperative day. The contrast material passed easily from the patient's stomach to the duodenum and no stenosis in the region of the anastomoses or leaks or fistulae appeared (Figure 4). A cholangiography was also performed through the T-tube. This showed a satisfactory flow through the patient's bile duct and an unobstructed passage of the contrast agent to his duodenum (Figure 5). The T-tube was removed the following day (21^st ^postoperative day). The patient was discharged on the 22^nd ^postoperative day in excellent general condition and has remained so to present date, five years later.

## Discussion

Due to its retroperitoneal location, injuries of the duodenum are uncommon [1]. However, this location renders it inaccessible and consequently patients with injuries to the duodenum after a blunt abdominal trauma are diagnosed late, although more apparent injuries to other organs or vessels are addressed [3-5]. The duodenum is only mobile at the pylorus and its fourth part. It shares its blood supply with the pancreas and, if its relation to the bile duct is taken into account, the high difficulty in suturing or resecting a segment of the duodenum, especially when the traumatic lesion involves its second part [1], is easily apparent.

Disruption of the duodenum by blunt force can occur either by crushing the duodenum against the rigid vertebral column (as from a direct blow to the abdomen), from the impact of shearing forces (as may occur during falls) or bursting energy (as with a seat belt injury) [5,6]. In our case, the most likely mechanisms of injury, based on the information from the site of the accident, were the effect of crushing and the impact of shearing forces.

Early diagnosis of a patient with a duodenal injury is critical and the time interval from injury to definite treatment influences morbidity and mortality from this injury. An 11% mortality rate in patients who underwent an operation less than 24 hours after an injury increases up to 40% in those who were operated on after 24 hours after being injured [7]. Information about the mechanism of injury and physical examination may arouse suspicion for duodenal injury. However, the retroperitoneal location of the duodenum may preclude early manifestation of injury and physical examination may be misleading with vague findings. Retroperitoneal duodenal perforation is usually subtle on presentation, although tachycardia, right upper-quadrant tenderness, vomiting and a progressive rise in temperature and heart rate are common findings in patients with this presentation [8]. When our patient was brought to the emergency room, he was haemodynamically stable, presenting with upper abdominal pain and tenderness on examination, and with haematemesis later on. Information about the mechanism of injury combined with the clinical findings aroused our suspicion of an intraabdominal organ injury; therefore, we proceeded promptly to the necessary laboratory and imaging studies.

A CT scan of the patient's abdomen with intraluminal and intravenous contrast is the diagnostic test of choice in stable patients with blunt abdominal trauma, and provides excellent anatomic detail of the retroperitoneum. However, CT scanning cannot always distinguish duodenal perforations from duodenal haematomas [9,10]. In our case report, the deterioration of the patient's clinical status including haematemesis and the inherent high suspicion of abdominal injury indicated the investigation of the intraperitoneal and retroperitoneal space with a CT scan. Although the CT scan did not show any duodenal disruptions, its findings combined with the clinical findings and the history of the accident increased our suspicion of a possible retroperitoneal duodenal injury.

A combined injury of the pancreas and duodenum has been regarded as a separate category of injury, with a particularly high mortality [11]. It has been suggested that even minor injuries to the pancreas increase rates of morbidity and mortality from associated duodenal injuries [11]. However, pancreatic lacerations that do not involve the major pancreatic duct and that spare the bile duct appear to have lower rates of morbidity and mortality [11]. In our case report, after investigation of the status of the patient's main pancreatic and bile ducts, we verified that the ducts were not involved.

Although a grading system has been devised to characterise duodenal injuries, it is less important than several simple aspects of the duodenal injury that better serve, from a practical point of view, the goal of definite treatment [12]. These aspects are the anatomical relation of the injury to the ampulla of Vater, the characteristics of the injury (simple laceration versus destruction of the duodenal wall), the involved circumference of the duodenum, the associated injury to the biliary tract, pancreas or major vascular injury, and the time elapsed until the patient receives definite treatment [12]. In our case report, these aspects were decisive for the characterisation of the patient's injury and surgical technique selection.

Several surgical techniques have been described for the adequate treatment of patients with duodenal injuries, according to location and type of injury. In our case report, where the second part of the patient's duodenum was completely transected, our choices for reconstruction were limited either to a primary end-to-end anastomosis or Roux-en-Y duodenojejunostomy with closure of the distal duodenal stump [2]. A primary end-to-end anastomosis was ruled out because of the difficult mobilisation of the duodenum at that particular part. Also, performing an anastomosis subjected to undue tension could result in anastomotic dehiscence and development of fistulae, intraabdominal abscesses or duodenal obstruction, not to mention that such a repair would necessitate an additional gastrojejunostomy. Considering that the technique of pedicled mucosal graft, using jejunum [13], ileum [14] or stomach island flap [15], has been suggested as a method of closing large duodenal defects, we decided that the duodenal continuity would be better restored interposing an intact pedicled loop (15 cm long) between the duodenal stumps. With this technique the restoration of the duodenal continuity is more physiological (especially in a teenager with a still developing body), the diameter of the graft was the same with the duodenum, there was no undue tension at the anastomotic sites, and the repair was technically easier. Except for the mild pancreatitis, the patient presented with no other postoperative complications and was discharged on the 22^nd ^postoperative day in excellent condition.

## Conclusions

The most important factors for the successful management of the patient with duodenal injury were the short time interval between injury and operation (four hours), the meticulous exploration and drainage of the retroperitoneal haematoma, the assessment of the pancreatic rupture and the verification that no associated injuries to the pancreatic duct, common bile duct and Vater's papilla had occurred. The technique that we used restored the physiological anatomical continuity of the patient's duodenum.

## Consent

Written informed consent was obtained from the patient for publication of this case report and accompanying images. A copy of the written consent is available for review by the Editor-in-Chief of this journal. The patient was an adult at the time of submission (21 years old), when he signed the consent form.

## Competing interests

The authors declare that they have no competing interests.

## Authors' contributions

AK was the attending surgeon and wrote the initial draft. NA assisted on the operation and collection of bibliographical data. SP wrote the final manuscript. CS assisted on selection of bibliographical references. TG is the head of the department. All authors have read and approved the final manuscript.
